# miR-143 or miR-145 overexpression increases cetuximab-mediated antibody-dependent cellular cytotoxicity in human colon cancer cells

**DOI:** 10.18632/oncotarget.7010

**Published:** 2016-01-25

**Authors:** Sofia E. Gomes, André E. S. Simões, Diane M. Pereira, Rui E. Castro, Cecília M. P. Rodrigues, Pedro M. Borralho

**Affiliations:** ^1^ Research Institute for Medicines (iMed.ULisboa), Faculty of Pharmacy, Universidade de Lisboa, Lisbon, Portugal

**Keywords:** miR-143, miR-145, cetuximab, ADCC, granzyme B

## Abstract

miR-143 and miR-145 are downregulated in colon cancer. Here, we tested the effect of restoring these miRNAs on sensitization to cetuximab in mutant KRAS (HCT116 and SW480) and wild-type KRAS (SW48) colon cancer cells. We evaluated cetuximab-mediated antibody-dependent cellular cytotoxicity (ADCC) and the modulation of signaling pathways involved in immune effector cell-mediated elimination of cancer cells. Stable miR-143 or miR-145 overexpression increased cell sensitivity to cetuximab, resulting in a significant increase of cetuximab-mediated ADCC independently of KRAS status. Importantly, HCT116 cells overexpressing these miRNAs triggered apoptosis in result of cetuximab-mediated ADCC, effected by peripheral blood mononuclear cells (*p* < 0.01). This was associated with increased apoptosis and caspase-3/7 activity, and reduced Bcl-2 protein expression (*p* < 0.01). In addition, caspase inhibition abrogated cetuximab-mediated ADCC in HCT116 cells overexpressing either miR-143 or miR-145 (*p* < 0.01). Furthermore, Bcl-2 silencing led to high level of cetuximab-mediated ADCC, compared to control siRNA (*p* < 0.05). Importantly, granzyme B inhibition, abrogated cetuximab-mediated ADCC, reducing caspase-3/7 activity (*p* < 0.01). Collectively, our data suggests that re-introduction of miR-143 or miR-145 may provide a new approach for development of therapeutic strategies to re-sensitize colon cancer cells to cetuximab by stimulating cetuximab-dependent ADCC to induce cell death.

## INTRODUCTION

Colon cancer is among the most frequent malignant diseases in Western industrialized countries, and one of the most common cancer types. Colon cancer is also highly ranked in incidence and cancer-related deaths worldwide [1], being the third most common cancer in men (746,000 cases, 10% of the total), second in women (614,000 cases, 9.2% of the total), and the fourth leading cause of cancer-related deaths in the world, accounting for 8.5% of cancer-related mortality [2]. Over the last ten years, the introduction of targeted agents in clinical practice has led to improvements in treatment of metastatic colon cancer. Among these, cetuximab is a partially humanized monoclonal antibody, raised against the epidermal growth factor receptor (EGFR), which abrogates EGFR signaling by impairing receptor dimerization, promoting receptor internalization and degradation, and by activating antibody-dependent cellular cytotoxicity (ADCC) [3, 4]. Despite therapeutic advances, the high rates of intrinsic and/or acquired drug resistance severely narrow clinical options for naïve and refractory patients, highlighting the need for novel, more effective therapeutic strategies. Although the targeting of EGFR pathways has been quite successful, mutations in KRAS carry a negative response predictive value of 99%, making it an ideal predictive biomarker for EGFR targeted therapy [5]. This dramatically reduces the therapeutic options for advanced colorectal cancer harboring KRAS mutations.

The discovery of microRNAs (miRNAs/miRs) provides a new hope for improving disease outcome and has gained increasing relevance as putative cancer therapeutics [6]. miRNAs are small non-coding RNAs, which post-transcriptionally regulate gene expression [7]. miRNAs undergo several bioprocessing steps until the mature miRNA, a 15–22 nt single-strand RNA, enters the cytoplasmatic multiprotein complex termed miRNA-induced silencing complex (miRISC). Mature miRNAs loaded onto miRISC are then able to bind to the 3′-untranslated region (3′-UTR) of target mRNAs carrying complementary sequences, thereby repressing gene expression [8]. Imperfect base pairing between miRNAs and mRNAs is a common event, and enables individual miRNAs to target a large, yet incompletely identified, set of genes. Consequently, the ability of a single miRNA to control several players of oncogenic signaling pathways deregulated in cancer may hold the key to therapeutic success [9–11].

Aberrant miRNA expression is widely reported in cancer [12, 13], including colon cancer [14–16], providing relevant insights into cancer tumorigenesis, diagnosis, prognosis, and therapy response [9]. Furthermore, miRNA modulation induces potent anticancer effects and sensitizes to anticancer drugs [17–21], displaying enormous cancer therapeutic potential. Importantly, in experimental cancer models, the delivery of anti-tumorigenic miRNAs, including miR-143 and miR-145, appears to be beneficial for cancer therapy [21, 22].

miR-143 and miR-145 are tumor suppressor miRNAs reported downregulated in several cancer types, including in colon cancer adenomas and carcinomas [17–19, 23]. These miRNAs are involved in the regulation of several cellular processes including proliferation, migration and chemoresistance [19, 24–26]. We have shown that miR-143 overexpression induces tumor cell sensitization to 5-fluorouracil [18]. In addition, miR-143 is also chemosensitizer to docetaxel in prostate cancer by targeting KRAS and subsequently targeting EGFR/RAS/MAPK pathway [27], while miR-145 inhibits EGFR mutant lung cancer cell growth, sensitizing to gifitinib [25]. Further, combined overexpression of miR-143 and miR-145 decreases squamous carcinoma proliferation while sensitizing to cisplatin, and also sensitizes colon cancer cells to 5-FU, irinotecan and oxaliplatin treatment [23]. The role of these miRNAs in EGFR signaling regulation and cetuximab outcome in colon cancer is beginning to emerge, but remains poorly explored. It was reported that EGFR signaling pathway suppressed miR-143/145 in colonic cells, while overexpression of these miRNAs suppressed EGFR-induced colon cancer cell growth [28]. Further, miR-143 down-regulation was correlated with poor prognosis in wild-type KRAS patients, but low levels of this miRNA did not display predictive value for response to EGFR-targeted agents [29].

In the current study, we evaluated the role of miR-143 or miR-145 in the modulation of response to cetuximab in colon cancer cells derived from human primary colon carcinomas (HCT116, SW480 and SW48) [30, 31], widely used for the study of cancer biology and cellular response to drugs, including ADCC [32, 33]. Our results showed that miR-143 or miR-145 overexpression increased the sensitivity of colon cancer cells to cetuximab by enhancing cetuximab-mediated ADCC and apoptosis. Therefore, we propose that restoration of these miRNAs contributes to cetuximab sensitization both in KRAS mutant cetuximab-resistant and in KRAS wild-type colon cancer cells.

## RESULTS

### miR-143 or miR-145 overexpression sensitizes colon cancer cells to cetuximab

**Figure 1:**
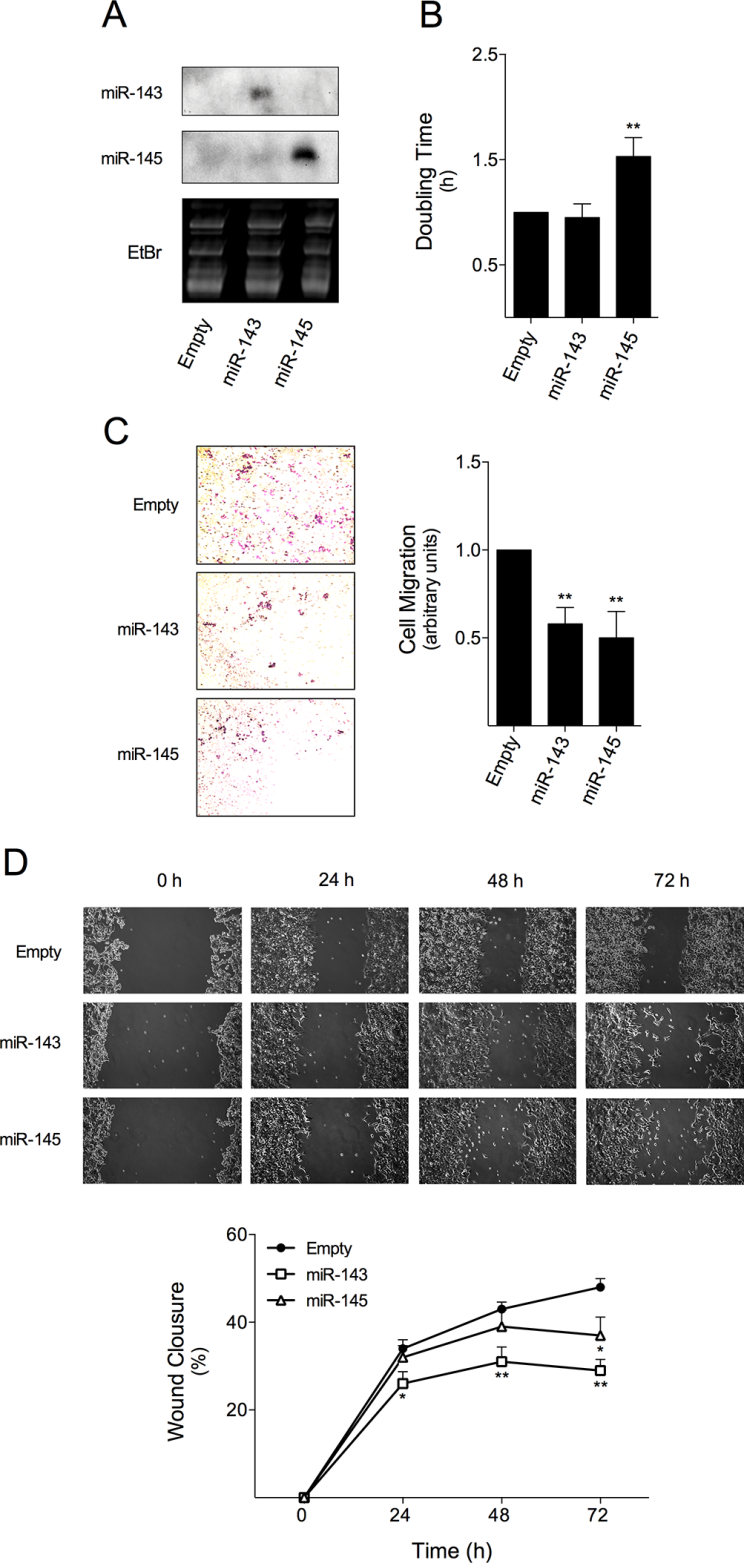
miR-143 or miR-145 overexpression reduces HCT116 colon cancer cell doubling time and migration miR-143 or miR-145 overexpressing cells were produced by transducing HCT116 cell line with viral particles containing MSCV-Neo constructs expressing miR-143, miR-145 or empty vector, as control. (**A**) miR expression was assayed by northern blot. Gel loading controls are shown from ethidium bromide (EtBr) staining of RNA. (**B**) HCT116-Empty, HCT116-miR-143, and HCT116-miR-145 cells were plated onto a 96-well E-Plate of xCELLigence System. Cell index was measured every 5 min for 24 h and used to plot and calculate cell doubling time. (**C**) Cell migration was assessed by transwell migration assay, with cells allowed to migrate for 9 h after cell platting; (**D**) and by wound healing assay at 24, 48 and 72 h after wound formation. Results are expressed as (B, C) mean ± SEM fold change to control cells, or (D) percentage of wound closure ± SEM, from at least three independent experiments. **p < 0.01 and *p < 0.05 from HCT116-Empty cells.

To gain insight into the biological effect of miR-143 or miR-145 in colon cancer cell response to cetuximab, HCT116 cells were transduced with retroviral particles containing MSCV-Neo constructs expressing miR-143 or miR-145, respectively, resulting in HCT116 overexpressing miR-143 (HCT116-miR-143) and miR-145 (HCT116-miR-145), and the respective Empty vector control cell line (HCT116-Empty). The overexpression of these miRNAs was confirmed by Northern blot (Figure 1A). Next, we evaluated the effect of miR-143 or miR-145 overexpression in HCT116 cell proliferation in real-time, using the xCELLigence system. We observed a significant increase in cell doubling time in HCT116-miR-145 cell line compared with Empty control cells, suggesting that miR-145 impaired HCT116 cell proliferation (*p* < 0.01) (Figure 1B). In contrast, miR-143 overexpression did not alter cell doubling time. In addition, cell migration was significantly decreased in miR-143 or miR-145 transduced cells as compared to Empty control cells. In this regard, HCT116-miR-143 and HCT116-miR-145 cells displayed a 40 and 50%, reduction in transwell migration through 8 μM polycarbonate membrane filter, respectively, compared to HCT116-Empty cells (*p* < 0.01) (Figure 1C). In addition, wound healing assays confirmed these effects, since HCT116-miR-143 cells displayed a 30 and 40% reduced migration, respectively at 48 and 72 h, compared to HCT116-Empty control cells (*p* < 0.01); HCT116-miR-145 cells displayed nearly 20% reduced cell migration at 72 h compared to HCT116-Empty control cells (*p* < 0.05) (Figure 1D).

**Figure 2:**
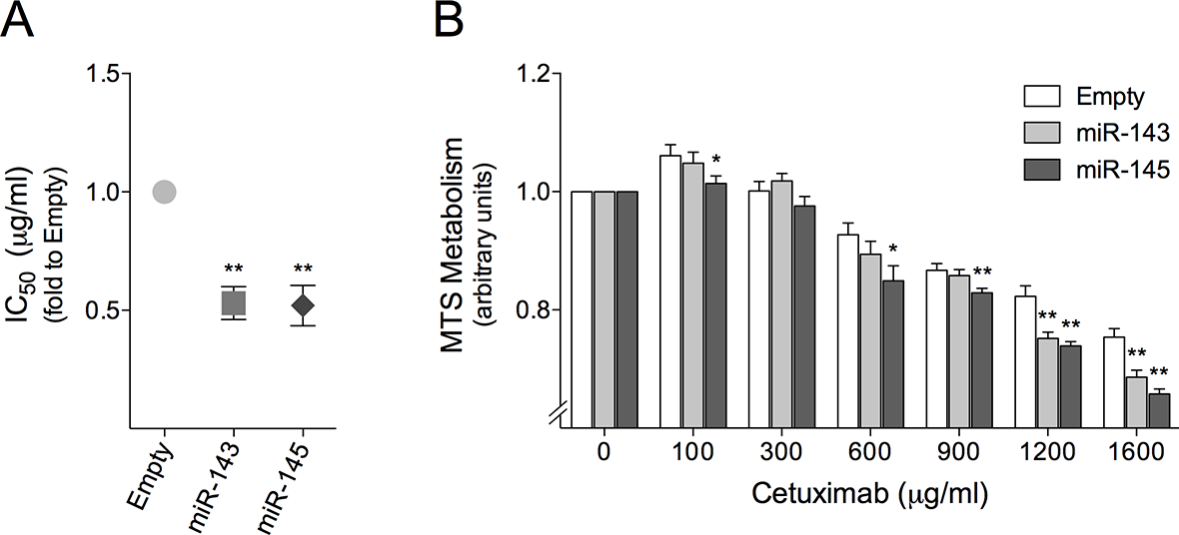
miR-143 or miR-145 overexpression sensitizes HCT116 mutant KRAS colon cancer cells to cetuximab HCT116-Empty, HCT116-miR-143 and HCT116-miR-145 stably transduced cells were plated onto a 96-well E-Plate of xCELLigence System. 24 h after plating cells were exposed to 0-1600 μg/ml cetuximab, and allowed to growth for 72 h. (**A**) Cell index was measured every 5 min, which allowed IC_50_ determination from the time of incubation. (**B**) Cell viability was also evaluated by MTS metabolism assay. The results are expressed as (A) the mean ± SEM fold change to control cells, or (B) the mean ± SEM fold change to respective untreated cells, from at least three independent experiments. **p < 0.01 and *p < 0.05 from HCT116-Empty cells.

We next investigated whether miR-143 or miR-145 overexpression could alter the sensitivity of HCT116 cells to cetuximab therapy. For this purpose, miR sensitization effects were assessed by calculating IC_50_ values for cetuximab using the xCELLigence system. Cetuximab showed a higher growth-inhibitory effect on cells overexpressing miR-143 or miR-145, with IC_50_ values of 832,22 and 668,42 μg/ml, respectively, compared to Empty control cells which displayed an IC_50_ of 1719,66 μg/ml (Table 1). These data clearly show that miR-143 or miR-145 overexpression in HCT116 cells led to a reduction of the IC_50_ value of cetuximab of nearly 40% (*p* < 0.01) (Figure 2A), indicating that these miRNAs may be involved in HCT116 cell response to cetuximab. To further explore these effects, we next exposed our stable miR overexpressing cell model to 0-1600 μg/ml cetuximab for 72 h, and evaluated the effect of stable miR-143 or miR-145 in cell viability by MTS metabolism assay. Our results indicate that overexpression of miR-143 or miR-145 significantly sensitized HCT116 cells to cetuximab (Figure 2B). miR-143 overexpression significantly decreased cell viability for cetuximab concentrations higher than 1200 μg/ml (*p* < 0.01), while miR-145 overexpression had a similar sensitization effect for cetuximab concentrations higher than 600 μg/ml (*p* < 0.05), both compared to Empty control cells (Figure 2B).

**Table 1:** Cetuximab IC_50_ in HCT116 colon cancer cells

HCT116 cell line	IC_50_ (μg/ml)
Empty	1719.66 ± 411.01
miR-143	832.22 ± 85.67
miR-145	668.42 ± 45.07

72 h IC_50_ values were calculated for cetuximab

We further ascertained if the role of miR-143 or miR-145 on increasing cetuximab sensitivity also occurs in KRAS wild-type SW48 colon cancer cells, which are sensitive to cetuximab. For this purpose, SW48 cells were stably transduced with the same retroviral particles used to generate HCT116 stable miRNAs overexpressing cells, resulting in cells overexpressing miR-143 (SW48-miR-143) and miR-145 (SW48-miR-145), and the respective Empty vector control cell line (SW48-Empty). miRNA expression was confirmed by Northern blot (Figure S1A). Next, SW48-derived cells were treated with increasing concentrations of cetuximab for 72 h, and cell viability was evaluated by MTS assay. Exposure of SW48-Empty cells to cetuximab resulted in an inhibition of cell viability within the entire range of concentrations tested. Importantly, overexpression of miR-143 and miR-145 significantly reduced cell viability of cells exposed to cetuximab concentrations higher than 1 μg/ml (*p* < 0.01) (Figure S1B). The results obtained suggest that miR-143 or miR-145 overexpression modulates cetuximab cell sensitivity independently of KRAS mutation status.

### miR-143 or miR-145 overexpression sensitizes colon cancer cells to cetuximab by increasing antibody-dependent cellular cytotoxicity

In addition to blocking EGFR signaling pathway, cetuximab also promotes cell death through ADCC [3]. Regarding ADCC-mediated effect, the constant fragment (Fc) domain of cetuximab recruits immune effector cells, such as monocytes, macrophages, lymphocytes and natural killer (NK) cells, which express Fc receptors (FcγRs), thus triggering their cytolytic activity mediated by perforins and granzymes, or by the Fas ligand (FasL) and tumor necrosis factor–related apoptosis-inducing ligand (TRAIL) [34–36]. Importantly, it is suggested that ADCC is a major *in vivo* action mechanism of therapeutic monoclonal antibodies (mAbs) [37], implying that cetuximab-induced ADCC may be an important mechanism of action of this mAb.

**Figure 3:**
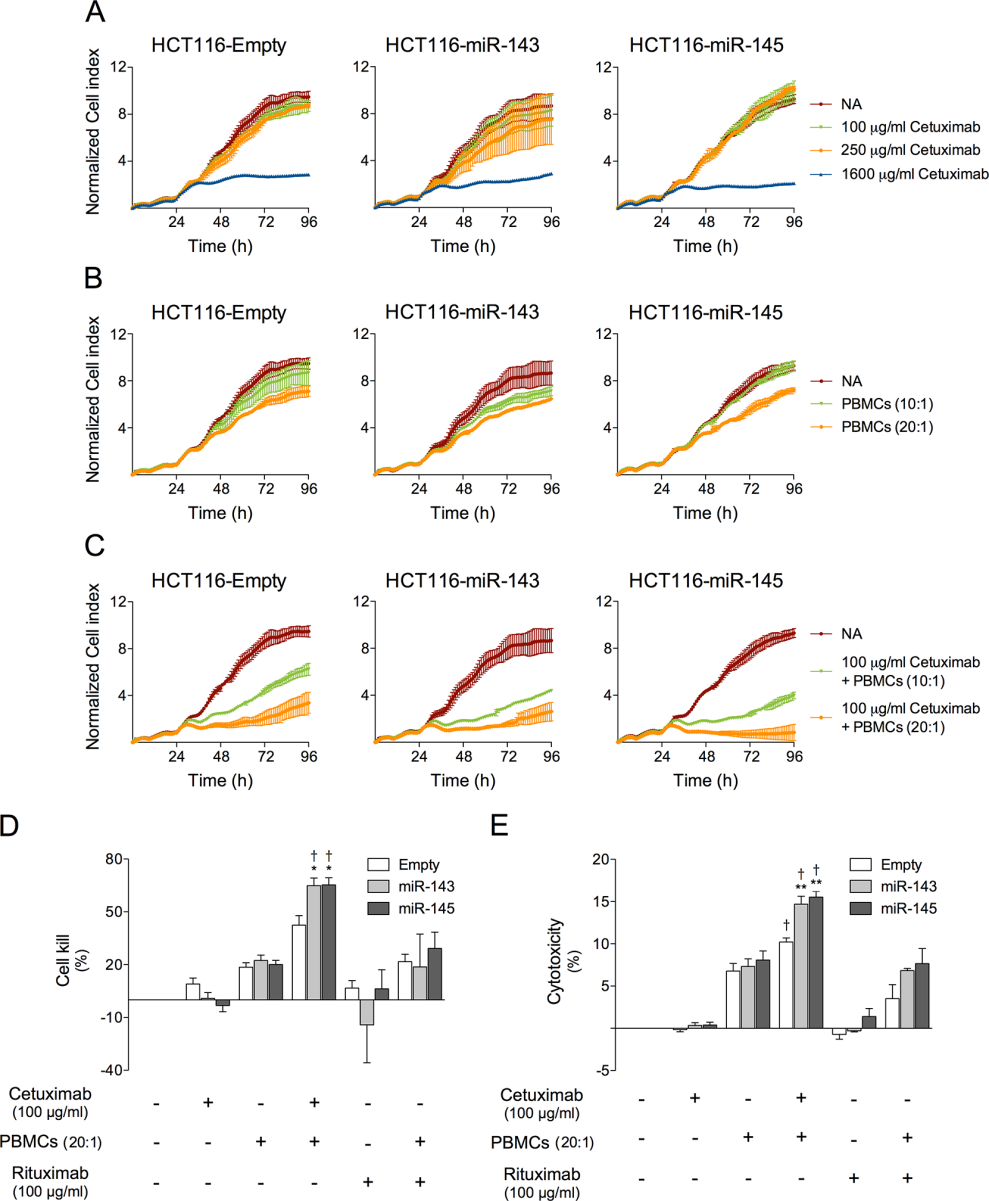
miR-143 or miR-145 overexpression increases cetuximab-mediated ADCC in HCT116 cells HCT116-Empty, HCT116-miR-143 and HCT116-miR-145 cells were plated on 96-well E-Plate and used on xCELLigence System, allowed to grow for 96 h. Cells were grown in medium alone or treated with increasing concentrations of cetuximab, and or PBMCs. (**A**) Red (NA) represents cells grown in medium alone, green represents growth with 100 μg/ml cetuximab, orange with 250 μg/ml cetuximab, and blue with 1600 μg/ml cetuximab. (**B**) Cells were grown in medium alone (red), or treated with PBMCs at (10:1) green or (20:1) orange. (**C**) Cells were grown in medium alone (red), or treated with 100 μg/ml cetuximab and PBMCs at (10:1) green or (20:1) orange. Cell index values were normalized at the time of the addition. Normalized cell index values are plotted in 1 h increments as the average of two replicates together with standard deviation. (**D**) Quantification of normalized cell index was performed at 72 h, by measuring the change in area under the curve compared to non-treated controls, and are presented as percentage of cell kill for 100 μg/ml cetuximab treatment or 100 μg/ml rituximab (control antibody), alone or with PBMCs (20:1). (**E**) Quantification of cytotoxicity was performed at 48 h, by measuring the amount of LDH released into the culture supernatant, and is presented as percentage of cytotoxicity for 100 μg/ml cetuximab treatment or 100 μg/ml rituximab (control antibody), alone or with PBMCs (20:1), compared with non-treated controls. The results are expressed as the mean ± SEM, from at least three independent experiments. **p < 0.001 and *p < 0.01 from respective HCT116-Empty treated cells; ^†^ *p* < 0.01 from the respective cell line treated with rituximab and PBMCs.

To evaluate the role of miR-143 or miR-145 in cetuximab-mediated ADCC, we used our cell model HCT116-miR-143, HCT116-miR-145 and HCT116-Empty cell lines (target cells) and peripheral blood mononuclear cells (PBMCs) isolated from human healthy donors (effector cells). Tumor cell death by cetuximab-mediated ADCC was evaluated in real-time using the xCELLigence system. As expected, an increase in cell index as a function of time in culture was seen for all HCT116-derived cells growing in media alone for 96 h (Figure 3A). Further, treatment of HCT116 cells with 100 or 250 μg/ml cetuximab almost did not change the cell index values compared to untreated cells up to 72 h of exposure, regardless of miR-overexpression. In contrast, cetuximab at 1600 μg/ml led to a significant reduction in cell index values, to a higher extent in HCT116-miR-143, HCT116-miR-145 compared to HCT116-Empty, leading to a lower area under the growth curve (AUC) (Figure 3A).

Evaluation of ADCC was next performed using 100 μg/ml cetuximab and two different effector (E) to target (T) cell ratio (E:T), 10:1 and 20:1, which alone did not induce high levels of cell killing (Figure 3B). Importantly, 100 μg/ml cetuximab concentration, which we previously found ineffective in reducing HCT116 cell viability (Figure 2), when combined with effector cells, significantly decreased cell index values, resulting in higher growth-inhibitory effects on miRNA overexpressing cell lines, HCT116-miR-143 and HCT116-miR-145, compared to HCT116-Empty vector cells (Figure 3C). These effects were visible at an E:T ratio of 10:1, but were more pronounced at E:T ratio of 20:1, hence this latter ratio was chosen for further experiments. As a quantitative indicator of target cell killing activity mediated by effector cells in the presence of cetuximab, we determined the AUC for 72 h of treatment, and calculated the percentage cell killing (Cell kill (%)), using the formula described in materials and methods section. Cell kill determined for target cells exposed to 100 μg/ml cetuximab alone, was less than 10% for all cell lines, while exposure to effector cells at 20:1 E:T ratio increased cell kill to ~ 20% in all cell lines, regardless of miRNA expression levels. Importantly, exposure to 100 μg/ml cetuximab and effector cells at 20:1 E:T, resulted in higher growth-inhibitory effects in HCT116-miR-143 and HCT116-miR-145, ~ 65% cell kill, compared to 42% cell kill in HCT116-Empty cells (*p* < 0.01). Rituximab was used as a control antibody, and its addition alone or together with effector cells at 20:1 E:T, did not significantly increase cell kill, indicating that ADCC is a mechanism of cell death induction for cetuximab, and that miR-143 and miR-145 are modulators of this mechanism of antibody-mediated cell death (*p* < 0.01) (Figure 3D).

In addition, under the same conditions, cetuximab treatment combined with effector cells significantly increased death of cells overexpressing miR-143 or miR-145 compared to Empty-vector control (*p* < 0.001), evaluated by LDH release assay. Similarly to xCELLigence assays, control antibody rituximab alone or together with effector cells did not significantly increase cytotoxicity in HCT116-miR-143 and HCT116-miR-145 (*p* < 0.01) (Figure 3E).

We next ascertained if miR-143 or miR-145 increased cetuximab-mediated ADCC also occurred in other colon cancer cell lines, including cetuximab-sensitive cells. For this purpose, SW48-derived cells (Figure S1A) were exposed to cetuximab and effector cells, and cetuximab-mediated ADCC was evaluated by xCELLigence and LDH release assays. As expected, our results demonstrated that SW48-derived cells treated with lower concentrations of cetuximab, 1 and 10 μg/ml, display reduced cell index values compared to untreated cells, with this reduction being more pronounced in SW48-miR-143 and SW48-miR-145, compared to SW48-Empty cells (Figure S2A). Moreover, the addition of cetuximab and effector cells at 6:1 E:T ratio, which alone did not alter the cell index (Figure S2B), resulted in a significant decrease in cell index values for 1 and 10 μg/ml cetuximab (Figure S2C). Importantly, higher growth-inhibitory effects were observed on SW48-miR-143 and SW48-miR-145, ~ 40% cell kill, compared to 29% cell kill in SW48-Empty cells (*p* < 0.01) (Figure S2D). Corroborating these results, LDH release was significantly increased in cells overexpressing miR-143 or miR-145 treated with cetuximab combined with effector cells, compared to Empty-vector control (*p* < 0.05) (Figure S2E). Confirming the specificity of cetuximab-mediated ADCC, no significant increase in cell kill and cytotoxicity was detected in SW48-derived cells following exposure to rituximab and effector cells.

We additionally validated the results obtained for KRAS mutant HCT116 cells in another KRAS mutant, cetuximab-resistant cell line, SW480. For this purpose, SW480 cells were stably traduced with retroviruses overexpressing miR-143 or miR-145, and the control Empty vector, similarly to HCT116 and SW48 cell models, in order to generate SW480 cells overexpressing miR-143 (SW480-miR-143), and miR-145 (SW480-miR-145), and the respective Empty-vector control (SW480-Empty). Northern blot was performed to confirm miRNA expression levels (Figure S3A). Next, to evaluate cetuximab-mediated ADCC, cells were treated with 100 μg/ml cetuximab, and/or with PBMCs, as effector cells. The results obtained demonstrated that treatment of these cetuximab-resistant cells with 100 μg/ml cetuximab did not induce changes in the cell index values compared to untreated cells (Figure S3B). Similarly to HCT116-derived cells, exposure of SW480-derived cells to PBMCs at a 20:1 E:T ratio did not induce high levels of cell killing (Figure S3C), while exposure to 100 μg/ml cetuximab and PBMCs, significantly decreased cell index values (Figure S3D), resulting in higher growth-inhibitory effects in SW480-miR-143 and SW480-miR-145 cells, ~ 50% cell kill, compared to 28% cell kill in SW480-Empty cells (*p* < 0.05). Treatment of SW480-derived cells with the control antibody rituximab, and PBMCs increased cell kill to an extent similar to that elicited by PBMCs (Figure S3E). In this context, miR-143 or miR-145 overexpression significantly increased cell death of SW480 cells exposed to cetuximab together with effector cells, and again, exposure to control antibody rituximab, and effector cells only increased cell cytotoxicity to a similar extent of that elicited by PBMCs (Figure S3F). Therefore, taken together, these results unveil the importance of miR-143 and miR-145 in modulating ADCC as a mechanism of cell death induced by cetuximab in presence of PBMCs.

In addition, we were also interested in evaluating if the role of miR-143 or miR-145 on increasing ADCC in colon cancer cells was cetuximab-specific. Therefore, we evaluated the effect of these miRNAs in modulating the sensitivity to another targeted agent that also induces ADCC. Trastuzumab is a monoclonal antibody used in the treatment of metastatic breast cancer, which targets HER2 receptor, leading to the inhibition of its downstream signaling pathway. Among the proposed mechanism of action of this agent, ADCC is suggested as a major mechanism of trastuzumab-mediated tumor cell death [37, 38]. Further, HCT116 cells have previously been reported to express HER2 receptor [39] and trastuzumab-mediated HER-2 inhibition failed to block HCT116 cell proliferation [40]. Therefore, HCT116-derived cells were exposed to trastuzumab and effector cells, and target cell death was evaluated. As expected, exposure to trastuzumab alone did not induce cell kill. However the addition of trastuzumab and effector cells significantly increased death of cells overexpressing miR-143 and miR-145, observed in both xCELLigence and LDH released assays (*p* < 0.01). Importantly, trastuzumab-mediated cytotoxicity observed was significantly higher compared to the cytotoxicity induced by the control antibody rituximab (*p* < 0.05), confirming the specificity of trastuzumab-mediated ADCC, and expanding to role of miR-143 and miR-145 in sensitization to ADCC, suggesting that this cellular effect may be in play independently of the therapeutic antibody used to elicit ADCC, further highlighting the relevance of these miRNAs as promising therapeutic agents (Figure S4).

### Apoptosis is increased in miR-143 or miR-145 overexpressing cells during cetuximab-mediated ADCC

**Figure 4:**
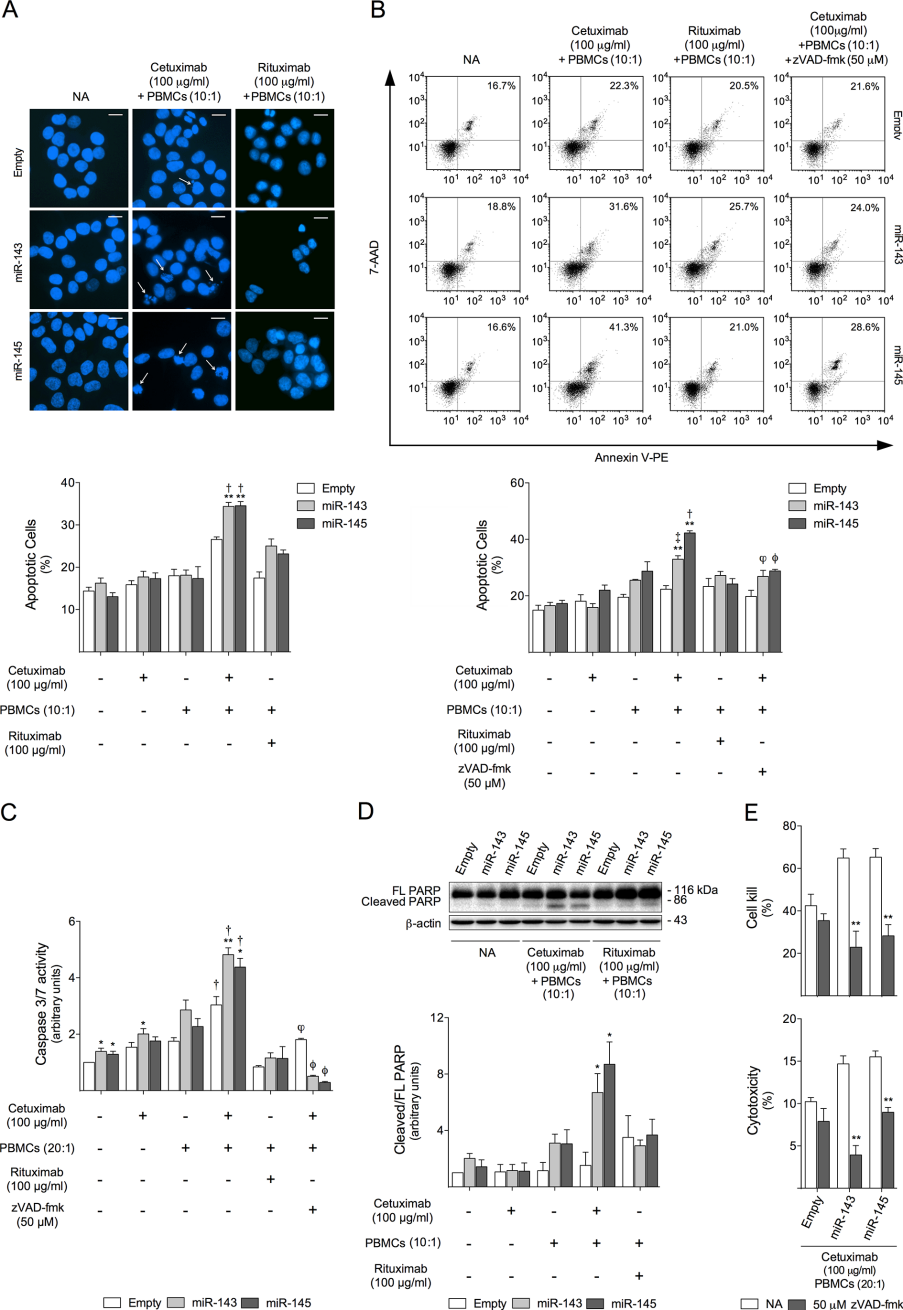
miR-143 or miR-145 overexpressing cells are more sensitive to cetuximab-mediated ADCC-induced apoptosis HCT116-Empty, HCT116-miR-143 and HCT116-miR-145 cells were exposed to 100 μg/ml cetuximab, 100 μg/ml rituximab or PBMCs (10:1 or 20:1) alone, or co-treatment of cetuximab or rituximab together with PBMCs (10:1 or 20:1), or vehicle (control). When indicated HCT116-derived cells were pretreated for 1 h with 50 μM z-VAD-fmk. (**A**) Nuclear morphology was evaluated by fluorescence microscopy after Hoechst staining, 24 h after treatment. Representative images of Hoechst staining at 400x magnification are presented. Arrows indicate nuclear fragmentation and chromatin condensation. Scale bar corresponds to 15 μm. (**B**) Apoptosis was quantified by flow cytometry using Guava Nexin assay, 48 h after treatment. (upper panel) Representative flow cytometry plots and percentage of cells positive for Annexin V and/or 7-AAD of one representative experiment. (lower panel) Quantification of apoptotic cells, positive for Annexin V and/or 7-AAD. (**C**) Caspase-3/7 activity was determined at 16 h after treatment, and (**D**) PARP cleavage was evaluated by immunoblot at 48 h after treatment. (**E**) Cetuximab-mediated ADCC was assayed using the xCELLigence system (upper panel) and LDH release assay (lower panel) as described above. Results are expressed as (A) percentage of apoptotic cells per field ± SEM, (B) percentage of apoptotic cells ± SEM, (C, D) mean ± SEM fold change to untreated control cells, and (E) mean ± SEM, from at least three independent experiments. FL, full lengh. **p < 0.001 and *p < 0.01 from respective HCT116-Empty treated cells; ^†^*p* < 0.01 and ^‡^*p* < 0.05 from the respective cell line treated with rituximab and PBMCs; ^†^*p* < 0.01 and ^†^*p* < 0.05 from respective cell line treated with cetuximab and PBMCs.

To investigate whether apoptosis was induced during cetuximab-mediated ADCC, we performed nuclear morphology evaluation studies by fluorescent microscopy following Hoechst staining. Our results show that overexpression of miR-143 or miR-145 enhanced cetuximab-mediated ADCC-induced apoptosis, resulting in 23% increase in nuclear fragmentation and nuclear morphological changes characteristic of apoptosis, as compared to Empty control cells (*p* < 0.001) (Figure 4A). Confirming these observations, flow cytometry analysis of 7-AAD and Annexin-V staining indicated that treatment with cetuximab and PBMCs led to a significant increase in apoptotic cells of 32 and 42%, respectively in HCT116-miR-143 and HCT116-miR-145 compared to HCT116-Empty cells (*p* < 0.001) (Figure 4B). No significant increase in apoptosis was observed between cell lines treated with rituximab and PBMCs (Figure 4A and 4B). Apoptosis induction by cetuximab and PBMCs was further confirmed by caspase-3/7 activity assays, which indicated that exposure to cetuximab and PBMCs induced a significant increase in caspase-3/7 activity of 37 and 31%, respectively in HCT116-miR-143 and HCT116-miR-145, compared to HCT116-Empty control cells (*p* < 0.001 for HCT-miR-143 and *p* < 0.01 for HCT116-miR-145) (Figure 4C). Importantly, and confirming the specificity of cetuximab-mediated ADCC, no significant increase in caspase-3/7 activity was detected in our HCT116 cell model following exposure to rituximab and PBMCs (Figure 4C). In addition, the cleavage of the endogenous substrate of active caspase-3, PARP, was substantially increased up to ~ 4- and 6-fold, respectively in HCT116-miR-143 and HCT116-miR-145, compared to HCT116-Empty control cells (*p* < 0.01) (Figure 4D), further confirming that miR-143 and miR-145 overexpression increased apoptosis induced by cetuximab-mediated ADCC in HCT116 cells. Similarly, no significant increase in PARP cleavage was observed in HCT116-miR-143 and HCT116-miR-145 cells treated control antibody rituximab together with PBMCs (Figure 4D).

We next evaluated the involvement of caspase activation on cetuximab-mediated ADCC. For this purpose, we treated HCT116-derived cells with the pan-caspase inhibitor, z-VAD-fmk, before exposure to cetuximab and PBMCs, and confirmed a reduction in apoptosis (Figure 4B) and caspase-3/7 activity (Figure 4C). Importantly, caspase inhibition following exposure to cetuximab and effector cells, resulted in a significant cell kill reduction of 65 and 57%, respectively in HCT116-miR-143 and HCT116-miR-145, compared to the 17% reduction in HCT116-Empty cell line (*p* < 0.001) (Figure 4E, Upper Panel). Furthermore, LDH release following cetuximab and effector cells treatment was also significantly reduced in HCT116-miR-143 or HCT116-miR-145 cells pre-treated with z-VAD-fmk, respectively resulting in 73 and 42% reduction of the cytotoxic effect of cetuximab and PBMCs, respectively, compared to the 23% reduction observed in HCT116-Empty cells (*p* < 0.001) (Figure 4E, lower panel). Therefore, miR-143 or miR-145 overexpression triggered cetuximab-mediated ADCC inducing caspase-dependent apoptosis.

### Bcl-2 knock-down is involved in miR-143 or miR-145-increased cetuximab-mediated ADCC

In order to investigate the molecular mechanism of miR-143 or miR-145 activity in colon cancer and cetuximab sensitization, we examined the expression levels of Bcl-2, which was shown to be reduced following miR-143 overexpression [18, 19], and may be relevant in this cetuximab-sensitization context, due to role in apoptosis regulation.

**Figure 5:**
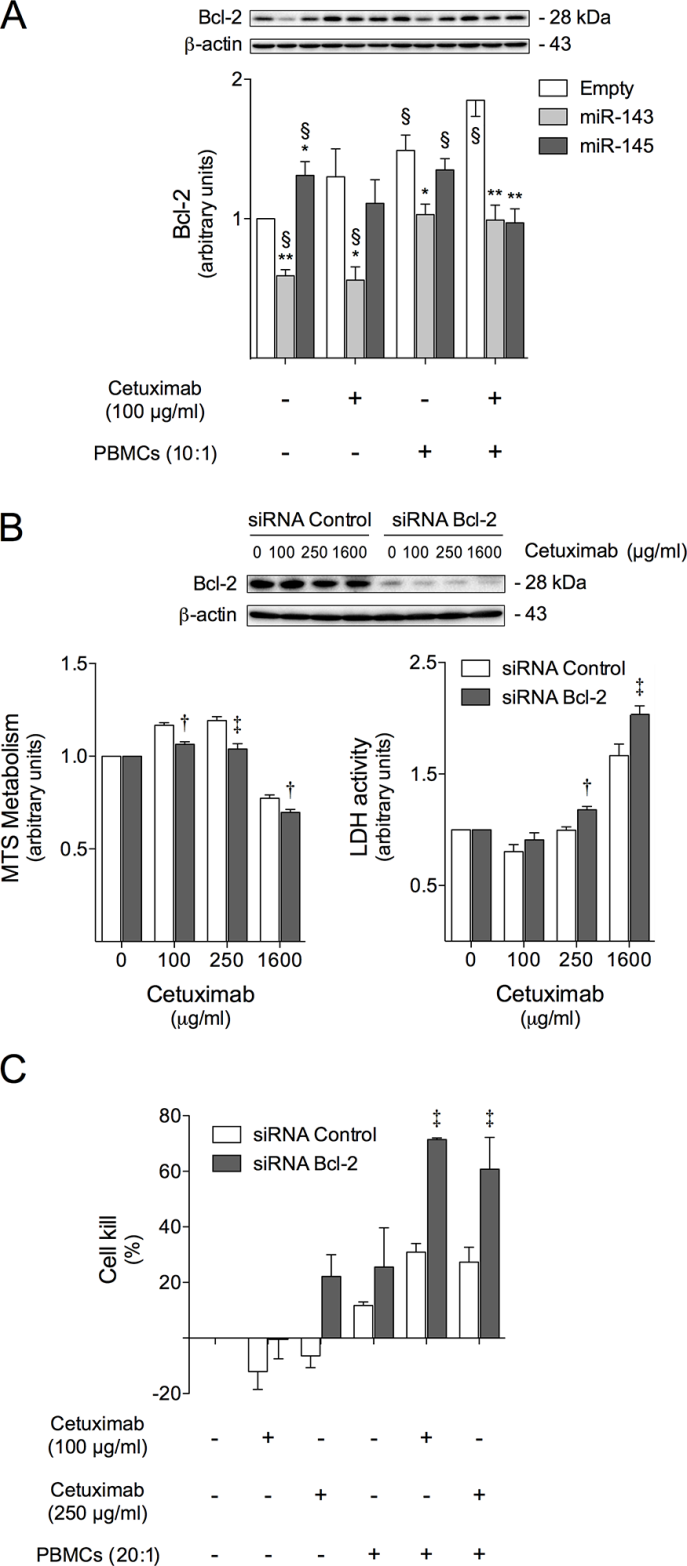
Bcl-2 is involved in cetuximab sensitization induced by miR-143 or miR-145 overexpression, increasing cell susceptibility to cetuximab-mediated ADCC (**A**) HCT116-Empty, HCT116-miR-143 and HCT116-miR-145 cells were exposed to 100 μg/ml cetuximab or PBMCs (10:1) alone, or co-treatment of cetuximab together with PBMCs (10:1), or vehicle (control) for 48 h to evaluate Bcl-2 protein expression, by immunoblot. HCT116 cells were transfected with Bcl-2 siRNA or siRNA control, and next treated with cetuximab for 72 h, and used for: (**B**) Bcl-2 protein expression evaluation by immunoblot (upper panel), and evaluation of cell viability and general cell death, respectively by MTS and LDH assay (lower panel); (**C**) cetuximab-mediated ADCC evaluation, where siRNA transfected cells were exposed to cetuximab and/or PBMCs (20:1) and the growth inhibitory effects were assayed using the xCELLigence system as described above. Quantification of growth inhibitory effects are presented as the percentage of cell kill for 100 μg/ml or 250 μg/ml cetuximab treatment, after 72 h of exposure. Representative blots from at least three independent experiments are shown. Results are expressed as (A) mean ± SEM fold-change to untreated control cells, (B) the mean ± SEM fold change to respective untreated cells, or (C) mean ± SEM, from at least three independent experiments. **p < 0.001 and *p < 0.01 from HCT116-Empty untreated cells; ^§^*p* < 0.01 from the respective HCT116-Empty untreated cells; ^†^*p* < 0.001 and ^‡^*p* < 0.01 from the respective HCT116-siRNA control transfected treated cells.

Interestingly, we found that miR-143 overexpression significantly decreased Bcl-2 protein steady-state expression. Our data demonstrated a 40% decrease in Bcl-2 steady-state levels in HCT116-miR-143 cells, compared to HCT116-Empty cells (*p* < 0.01) (Figure 5A, lane 2 *versus* 1). Further, following cetuximab exposure, Bcl-2 reduction in HCT116-miR-143 cells was even more pronounced, leading to an approximate 60% reduction as compared to HCT116-Empty cells (Figure 5A, lane 5 *versus* 4) (*p* < 0.01). In contrast, Bcl-2 steady-state expression levels in HCT116-miR-145 cells only slightly decreased following cetuximab treatment, which may be attributable to the fact that Bcl-2 is a miR-143 direct target [41]. Curiously, following exposure to cetuximab and/or PBMCs, Bcl-2 protein steady-state levels significantly increased in control cells. Interestingly, this effect was abrogated in cells overexpressing miR-143 or miR-145, in which Bcl-2 levels were reduced in almost 50% compared to empty control cells (*p* < 0.01) (Figure 5A, lanes 11 and 12 *versus* 10).

To explore whether increased sensitivity to cetuximab-mediated ADCC in HCT116 cells is regulated by Bcl-2 protein levels, Bcl-2 expression was efficiently knocked-down in HCT116 cells by transfecting a specific Bcl-2 siRNA (and a non-targeting control siRNA) (Figure 5B, upper panel). Subsequently, cells with Bcl-2 (and control) silencing were exposed to cetuximab and/or PBMCs. Our results from cytotoxicity assays showed that Bcl-2 silencing slightly reduced HCT116 cell viability and increased general cell death following cetuximab exposure at 250 μg/ml and 1600 μg/ml (*p* < 0.01) (Figure 5B, lower panel), compared with siRNA control transfected cells. No significant effect on cell death was observed following 100 μg/ml cetuximab treatment. Importantly, Bcl-2 silencing induced over 2-fold increase in cetuximab-mediated ADCC in the presence of PBMCs compared to control cells, as evidenced by a 70 and 60% cell kill, respectively for 100 μg/ml and 250 μg/ml cetuximab (*p* < 0.01), compared with control siRNA transfected cells, which displayed only 30% cell kill upon exposure to 100 or 250 μg/ml cetuximab (Figure 5C).

### Granzyme B activity is involved in miR-143 or miR-145-increased cetuximab-mediated ADCC

**Figure 6:**
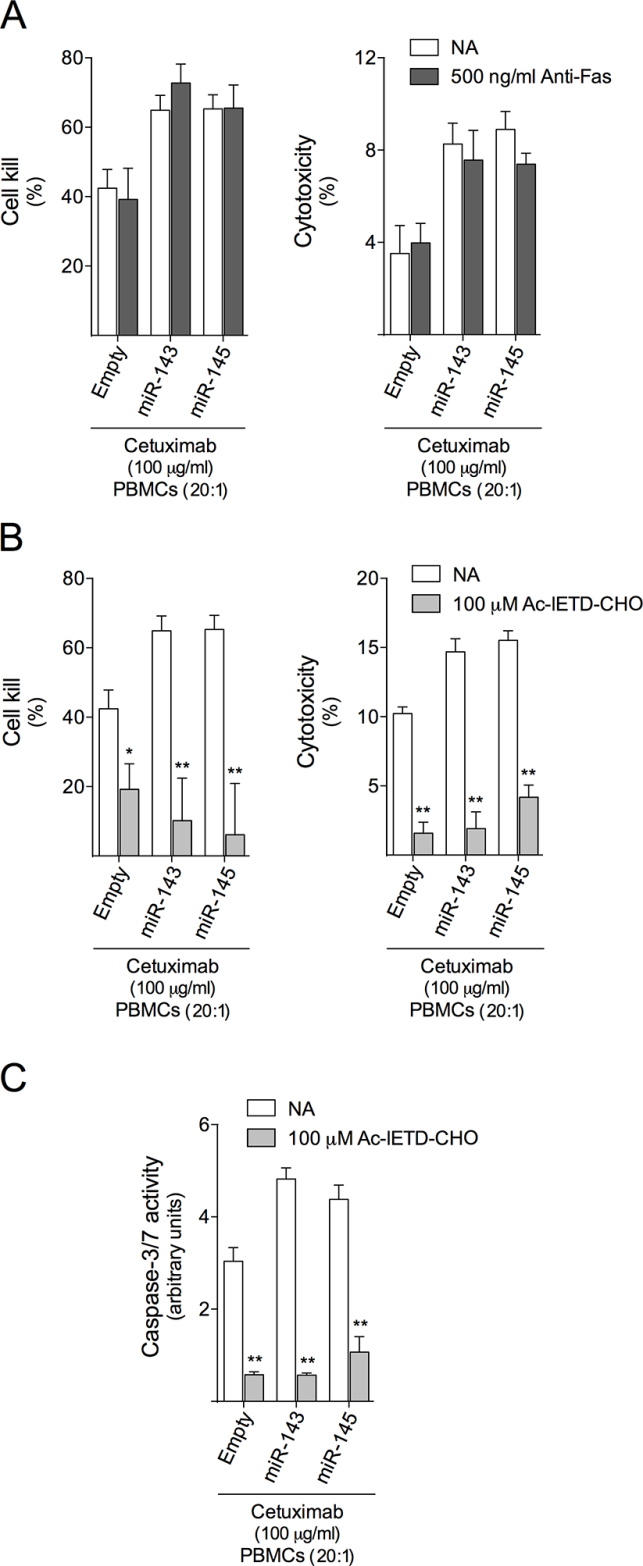
Granzyme B inhibition abrogates cetuximab-mediated ADCC in HCT116 cells HCT116-Empty, HCT116-miR-143 or HCT116-miR-145 cells were exposed to 100 μg/ml cetuximab or PBMCs (20:1) alone, or co-treatment of cetuximab together with PBMCs, or vehicle (control). Cetuximab-mediated ADCC was evaluated using the xCELLigence system and LDH release assay in (**A**) cells pre-treated for 2 h with 500 ng/ml of neutralizing antibody to Fas (ZB4), or in (**B**) cells exposed to PBMCs pretreated for 30 min with 100 μM Ac-IETD-CHO. (**C**) Caspase -3/7 activity was determined as described above. Results are expressed as (A, B) mean ± SEM, or (C) mean ± SEM fold-change to respective untreated cells. **p < 0.001 and *p < 0.05 from the respective untreated cells.

To further identify the mechanism underlying cetuximab-mediated ADCC in our cell model, we evaluated the signaling pathways mediating these effects. Effector cells mainly induce apoptosis in target cells by two mechanisms: death receptor-mediated (Fas-L- and TRAIL-mediated); and perforin/granzyme-mediated. Both pathways can directly activate caspase cascades, leading to target cell apoptosis. Therefore, we tested our HCT116 cell model for sensitivity to apoptosis triggered by Fas- or granzyme B-activated signaling pathways. In order to evaluate the involvement of Fas death-receptor signaling pathway in this context, we inhibited Fas receptor in target cells using a Fas neutralizing antibody. Our results showed that Fas blocking did not alter cetuximab-mediated ADCC (Figure 6A). Importantly, inhibition of granzyme B in PBMCs almost completely abrogated miR-143 or miR-145 increase in cetuximab-mediated ADCC in HCT116 cells (*p* < 0.001) (Figure 6B). Interestingly, this inhibition also occurred in empty control cells (*p* < 0.05). Therefore, our data clearly shows that granzyme B plays a key role in effecting cell death induced by cetuximab-mediated ADCC effected by PBMCs in HCT116 cells.

Activation of caspase-mediated apoptosis has been reported to be a hallmark of both Fas- and granzyme B-mediated cytotoxicity. Considering the lack of involvement of Fas in mediating cell death effected by PBMCs following cetuximab treatment, we next evaluated if caspase-dependent apoptosis resulting from cetuximab-mediated ADCC was dependent on granzyme B activity. To test this hypothesis, we exposed our HCT116 cell model to cetuximab and PBMCs previously incubated with granzyme B inhibitor, Ac-IETD-CHO, and evaluated caspase-3/7 activity. Our data confirmed that caspase-3/7 activity was almost completely abrogated, clearly confirming the involvement of granzyme B in the induction of caspase-dependent apoptosis induced by cetuximab-mediated ADCC. (Figure 6C).

## DISCUSSION

The heterogeneity of colon cancer contributes to several mechanisms of therapy resistance. Despite the fact that EGFR-targeted therapies, such as cetuximab, have been approved for clinical use only a small percentage of patients benefit from these therapeutic agents [42]. Therefore, identification of novel approaches to tackle cetuximab resistance would be key to sensitize cells to this therapy. Importantly, miRNAs have emerged as a great promise to make tumors responsive to chemotherapeutics, since miRNAs are able to target multiple proteins involved in different signaling pathways that are often compromised in cancer [10].

In this study, we observed that miR-143 or miR-145 overexpression reduced cell proliferation and migration of mutant KRAS HCT116 colon cancer cells, and sensitized both mutant KRAS (HCT116 and SW480), as well as wild-type KRAS (SW48) cells to cetuximab. miR-143 and miR-145 are co-transcribed miRNAs that have been widely studied as potential tumor suppressors. They were first reported as downregulated in colon cancer [43], and subsequently also in other solid tumors of epithelial origin [44–46]. Additionally, several functional studies have demonstrated that enforced ectopic expression of these miRNAs reduced proliferation, increased apoptosis and/or suppressed tumor-forming ability of diverse cancer cell types *in vitro* and *in vivo* [17–19, 47]. In addition, it was reported that decreased miR-143 expression might contribute to the pathogenesis of colon cancer by up-regulating KRAS expression [48], and miR-143 down-regulation correlates with poor prognosis in wild-type KRAS patients [29]. Similarly, miR-145 is also implicated in Ras/MAPK signaling pathway by targeting RREB1 protein, which negatively regulates the miR-143/145 promoter and potentiates signaling through Ras effector pathway [49]. Considering that the presence of mutations in *KRAS* gene renders tumor cells inherently resistant to anti-EGFR therapy, our results indicate that miR-143 or miR-145 may be key players in mitigating cetuximab resistance in mutant KRAS colon cancer cells.

According to the literature, the direct inhibition of cell growth mediated by cetuximab is limited, and requires a relative high dose of antibody [3, 50]. However, this monoclonal antibody is able to eliminate EGFR-expressing tumor cells via ADCC at much lower concentrations [3, 51], suggesting ADCC as an important antitumor mechanism of cetuximab. The interaction of the antigen-binding fragment (Fab) of cetuximab with EGFR together with the interaction of the Fc portion of cetuximab with FcγRs expressed by effector cells leads to ADCC initiation. In result, immune cells eliminate antibody-coated tumor cells [52]. The importance of ADCC for the *in vivo* effect of monoclonal antibodies is evident from previous studies showing loss of efficacy of these agents in FcRγ^−/−^mice that are deficient in activating Fc receptors [37]. In addition, FcγRIIa and FcγRIIIa gene polymorphisms have been involved in increased ADCC-related therapeutic response to cetuximab in early-stage [53] and metastatic colon cancer patients [54, 55].

In this study, we demonstrated that miR-143 or miR-145 overexpression increased the growth inhibitory effect of cetuximab in cetuximab-resistant HCT116 colon cancer cells, upon exposure to high doses of cetuximab (Figure 2). On the other hand, the combination of cetuximab and effector cells resulted in a significant increase of cytotoxicity in cetuximab-resistant (HCT116 and SW480) and -sensitive (SW48) colon cancer cells overexpressing miR-143 or miR-145, at clinically achievable cetuximab doses (Figure 3, S2 and S3). This suggests that these miRNAs potentiate the antitumor effects of cetuximab, which are likely based on the engagement of immune effector mechanisms, in particular ADCC, independently of KRAS mutation status. Considering that under the current clinical dosing regimen, cetuximab average steady state plasma concentration is estimated to be around 100 μg/ml [56, 57], our results indicate that the contribution of these miRNAs to cetuximab sensitivity in mutant KRAS HCT116 and SW480, and wild-type SW48 colon cancer cell lines could be relevant for future clinical studies in advancing the understanding of miRNA modulation as putative therapeutic approaches in colon cancer. Interestingly, we found that miR-143 or miR-145 are also able to increase trastuzumab-mediated ADCC (Figure S4), expanding the therapeutic relevance of these miRNAs and demonstrating that their effect is not restricted to cetuximab-mediated ADCC.

Activation of immune cells, such as natural killer (NK) cells, cytotoxic T lymphocytes and monocytes, through FcγR results in target cell killing via ADCC, which leads to tumor cell apoptosis [58, 59]. Our results suggest that HCT116-miR-143 and HCT116-miR-145 cells are more sensitive to apoptosis elicited by cetuximab-mediated ADCC, displaying increased caspase-3/7 activity, as compared to control cells (Figure 4). Importantly, caspase inhibition abrogated cell killing induced by effector cells and cetuximab in HCT116 cells overexpressing miR-143 or miR-145 (Figure 4), confirming the involvement of caspases in cetuximab-mediated ADCC. This increased apoptosis could be explained in part by the reduced Bcl-2 levels in miR-143 and miR-145 overexpressing cells compared to empty control cells following cetuximab and PBMCs exposure, increasing the susceptibility of cells to effector cell-mediated apoptosis. This is in agreement with our previous results, which demonstrated that miR-143 overexpression reduces Bcl-2 expression *in vitro* [18], and reduces colon cancer tumor xenograft growth displaying higher levels of tumor cell apoptosis with decreased steady-state levels of Bcl-2 [19]. Importantly, Bcl-2 and other anti-apoptotic proteins reported overexpressed in several cancers are associated with the development of resistance to anticancer agents [60, 61], highlighting the therapeutic potential of targeting Bcl-2 in cancer treatment.

Cytotoxic effector cells kill their target cells through the death receptor pathway (Fas/FasL or TRAILR/TRAIL) and/or the granule secretory pathway. In the first case, the engagement of death receptors by their cognate ligands results in activation of caspase proteolytic cascade [62]. In the second case, granzymes, such as granzyme B, are released in the intercellular space between target and effector cells, and enter the target cell via perforin-dependent or -independent manner. Once inside the target cell, granzyme B can induce apoptosis by targeting both cytosolic and nuclear substrates. The most well-known function of granzyme B is the direct cleavage of caspase-3 and/or Bid. Bid cleavage produces truncated Bid, which triggers the disruption of the mitochondrial membrane, resulting in caspase activation and release of proapoptotic proteins [63]. To better understand the involvement of both cytotoxic mechanisms in cetuximab-mediated ADCC triggered by PBMCs in HCT116 cells, we blocked Fas/FasL pathway and granzyme B-mediated, and evaluated their effect in cell killing. Inhibition of Fas did not alter the cytotoxicity induced by effector cells and cetuximab in our cell model (Figure 6). However, in marked contrast, granzyme B inhibition almost completely abrogated cell death as well as caspase-3/7 activity induced by effector cells in presence of cetuximab. This suggests that the granule secretory pathway is involved in the cytotoxicity resulting from cetuximab-mediated ADCC (Figure 6). Granzyme B-induced mitochondrial cell death was reported to be inhibited by Bcl-2 overexpression [64, 65], which blocks the release of proapoptotic proteins that suppress caspase inhibition, leading to a decreased sensitivity of target cells to immune cell-mediated cell death [66]. Indeed, reduced levels of Bcl-2 in our HCT116-miR-143 and HCT116-miR-145 overexpressing cells following treatment with effector cells and cetuximab (Figure 5), could promote the release of proapoptotic proteins, such as Smac/DIABLO and Omi/HtrA2 [66], thus contributing to granzyme B-induced apoptotic pathway by relieving caspase inhibition. Although the inhibition of granzyme B abrogated the cytotoxicity of effector cells and cetuximab regardless of miRNA expression levels, the extent of caspase and nuclear fragmentation inhibition was higher in cells overexpressing miR-143 or miR-145, which also display reduced Bcl-2 protein steady-state levels. Therefore, our data suggests that caspase activation in response to cetuximab and PBMCs proceeds through a granzyme B-dependent pathway, regulated by Bcl-2. Nevertheless, additional studies should be performed to deeply understand the involvement of these proteins in miR-143 or miR-145-mediated cetuximab sensitivity.

miRNAs function as genome master regulators, showing broad activity in several cancer types, and are able to sensitize tumor cells to therapy. This supports the evidence that miRNAs are promising candidates for the development of new approaches that could circumvent the resistance to chemotherapy and targeted agents, and could ultimately translate into improved care for colon cancer patients. Collectively, our data indicates that restoration of miR-143 or miR-145 reduces the aggressiveness of mutant KRAS HCT116 cells. In addition, forced expression of these miRNAs in both mutant and wild-type KRAS colon cancer cells increased their sensitivity to cetuximab by increasing cetuximab-mediated ADCC. Moreover, increased levels of effector cell-mediated caspase-dependent apoptosis were observed for mutant KRAS HCT116 miRNAs-overexpressing cells. Therefore, miR-143 and miR-145 modulation may constitute relevant candidates for future therapeutic interventions in combination with cetuximab treatment, and possibly also with other therapeutic antibodies with ADCC-inducing capability.

## MATERIALS AND METHODS

### Cell culture

HCT116, SW480 and SW48 human colon carcinoma cell line was purchased from European Collection of Cell Cultures (ECACC) (Porton Down, Salisbury, UK). HCT116 and SW48 cells were cultured in McCoy's medium supplemented with 10% fetal bovine serum (FBS) and 1% antibiotic/antimycotic solution. SW480 and Phi-Nx AMPHO cells (Indiana University National Gene Vector Repository (NGVB), Indianapolis, IN 46202, USA) were cultured in Dulbecco's modified Eagle's medium (DMEM) supplemented with 10% fetal bovine serum (FBS) and 1% antibiotic/antimycotic solution, all from Gibco, Life Technologies, Thermo Fisher Scientific, Massachusetts, USA. Cell lines were maintained at 37°C in a humidified atmosphere of 5% CO_2_.

### Plasmid constructs and virus packaging

MSCV-Neo retroviral expression constructs expressing miR-143 or miR-145 were kindly provided by Dr. Joshua Mendell, (Howard Hughes Medical Institute (HHMI), Johns Hopkins University, Baltimore, MD, USA) [47]. Retroviral particles were package by transfecting Phi-Nx AMPHO cells with MSCV-Neo vectors using Lipofectamine 2000 (Invitrogen, Thermo Fisher Scientific, Massachusetts, USA), according to the manufacturer's protocol. Retrovirus-containing supernatants were collected 48 h after transfection, filtered through a 0.22 μm filter, and stored at −80°C until use.

### Generation of stable miRNA overexpressing cells

To generate miR-143 or miR-145 stable overexpression cells, HCT116, SW480 and SW48 cells were seeded in 6-well plates at 3 × 10^5^ cells/well, and 24 h later, transduced with retroviral particles carrying MSCV-Neo constructs overexpressing miR-143, miR-145 or empty vector. Forty-eight hours after transduction, HCT116 and SW480 or SW48 transduced cell populations were selected with 1 mg/ml or 400 μg/ml neomycin (Gibco, Life Technologies, Thermo Fisher Scientific), respectively, for 2 weeks, and subsequently cultured as described above for parental HCT116, SW480 and SW48 cells.

### RNA extraction

Total RNA was extracted from cells with TRIzol reagent (Life Technologies, Thermo Fisher Scientific), according to the manufacturer's instructions. RNA concentration and purity was evaluated using a NanoDrop spectrophotometer (ND-1000; Thermo Fisher Scientific) [18].

### Evaluation of miRNA expression by northern blot

Northern blot experiments were performed as described in [67]. Briefly, 10 or 20 μg of total RNA isolated from HCT116, SW480 and SW48 cells with stable overexpression of miR-143 and miR-145 and empty control cells were loaded onto a 15% SequaGel (National Diagnostics, Atlanta, GA), electrophoresed, and transferred to nylon membranes at 15 V for 60 min using a Trans-Blot SD semidry transfer system (Bio-Rad Laboratories, Hercules, CA). RNA was cross-linked to membranes at 60°C for 2 h using freshly prepared cross-linking reagent [68]. Locked nucleic acid (LNA)-modified oligonucleotides specific for each miRNA were then hybridized to membranes using ULTRAhyb-Oligo buffer (Ambion, Life Technologies, Thermo Fisher Scientific). Locked nucleic acid (LNA)-modified oligonucleotides specific for each miRNA were purchased from Exiqon (Exiqon A/S, Vedbaek, Denmark) and labeled with digoxigenin (DIG), using an end tailing kit (Roche Applied Science, Indianapolis, IN). Sequences of the oligonucleotide probes were GAGCTACAGTGCTTCATCTCA (miR-143) and AGGGATTCCTGGGAAAACTGGAC (miR-145). After hybridization, membranes were washed with different-stringency buffer solutions and blocked in blocking buffer (DIG Wash and Block buffer set; Roche Applied Science) for 3 h at room temperature before incubation with an anti-DIG antibody at room temperature for 1 h. For miRNA detection, membranes were processed using a substrate solution (CSPD; Roche Applied Science), after washing in DIG washing buffer, visualized and acquired under ChemiDoc MP System (Bio-Rad). The relative intensities of RNA bands were detected using the Image Lab densitometric analysis program (version 4.1; Bio-Rad). As loading control, non-saturated images of ethidium bromide stained gel were taken before transfer to the membrane.

### siRNA transfections

HCT116 cells were seeded on 60 mm dishes at 2 × 10^6^ cells/dish. Twenty-four hours later, cells were transfected with 100 nM of Bcl-2 siRNA (#4392420) and a nonsilencing siRNA control (#4390844), using Lipofectamine 2000, according to the manufacturer's instructions (all from Invitrogen, Life Technologies, Thermo Fisher Scientific). Twenty-four hours after transfection, cells were detached and reseeded in 6-well plates at 3 × 10^5^ cells/well, and in 96-well plate or 96-well E-plate at 5,000 cells/well, respectively for the evaluation of protein expression, cell viability, and death.

### Human peripheral blood mononuclear cells isolation and handling

Peripheral blood mononuclear cells (PBMCs) were isolated from healthy volunteers with Ficoll-Paque Plus density gradient (GE Healthcare Bio-Sciences AB, Uppsala, Sweden) and used as effector cells, as described in [69]. After isolation, PBMCs were resuspended in RPMI 1640 medium supplemented with 10% FBS and 1% penicillin/streptomycin solution (all from Gibco, Life Technologies, Thermo Fisher Scientific), and left overnight at 37°C in a humidified atmosphere of 5% CO_2_. PBMCs were then counted by trypan blue exclusion assay, resuspended in cold freezing medium (FBS with 10% DMSO (Sigma-Aldrich, Munich, Germany) and stored as 4 × 10^6^ cells/vial, at −80°C until use. When needed, PBMCs were thawed, resuspended in culture medium, and incubated for 18 h at 37°C in a humidified atmosphere of 5% CO_2_.

### Cell treatments

Cetuximab and Rituximab were kindly provided by Hospital Santa Maria and Hospital São Francisco Xavier, Lisbon, Portugal. Dilutions of cetuximab and rituximab were freshly prepared in phosphate buffer saline (PBS). Twenty-four hours after plating, HCT116, SW480 and SW48 cells were incubated with either cetuximab or PBS (vehicle control), at the indicated concentrations and exposure times. For ADCC experiments HCT116, SW480 and SW48 cells (target cells) were incubated with cetuximab (1, 10, 100, 250 or 1600 μg/ml) or vehicle control, with medium with PBMCs (effector cells) at the indicated effector (E) to target (T) cell ratio (E:T), and cetuximab/rituximab with PBMCs in a similar E:T ratio. To inhibit granzyme B, PBMCs were pre-incubated with 100 μM of Granzyme B inhibitor II, Ac-IETD-CHO (Calbiochem, Merck Millipore, Massachusetts, USA) for 30 min at 37°C, before target cell treatment. For Fas neutralization, HCT116 cells were pre-treated with 500 ng/ml of Fas neutralizing antibody (ZB4) (Merck Millipore) for 2 h before the addition of cetuximab and/or PBMCs. To inhibit apoptosis, target cells were pre-incubated with 50 μM of Pan-Caspase inhibitor z-VAD-fmk for 1 h before cetuximab and/or PBMCs exposure.

### Transwell migration chamber assay

HCT116 cells with stable overexpression of miR-143 or miR-145, and empty control cells, were seeded on 6-well plates for 24 h, and next serum starved for 16 h by replacing the media with serum-free medium. Cell migration was evaluated using Neuro Probe 48-well Micro Chemotaxis Chamber (Neuro Probe Inc, Gaithersburg), as previously described by us [70]. Briefly, serum starved cells in serum-free medium were reseeded in the upper chamber at 1 × 10^4^ cells/well, on top of a polycarbonate membrane filter with 8 μM pore (Neuro Probe Inc). The lower chamber was filled with complete medium (10% FBS), as chemotractant. After 9 h, non-migrated cells at membrane top were removed, and migrated cells were fixed with cold methanol, stained with Giemsa (Merck Millipore), and counted. Images were captured by bright field microscopy, using a Axio Scope A.1 fluorescence microscope (Zeiss Axioskop; Carl Zeiss GmbH, Jena, Germany), under a 400 × magnification, using a DFC490 camera (Leica Microsystems AG, Heersbrugg, Switzerland) with the IM50 software for image acquisition (Leica Microsystems, version 1.20, Release 9). Number of migrated cells per well was determined by counting the total cells per well using Image J software (http://rsbweb.nih.gov/ij/).

### Wound healing assay

HCT116 cells with stable overexpression of miR-143 and miR-145 and empty control cells were seeded on 35 mm^3^ dishes at 3 × 10^6^ cells/dish, and allowed to grow to confluence. Next, “wounds” were performed using a sterile 10 μl tip, followed by 2 washes with culture medium. Cell migration to the “wound area” was evaluated and images captured at 24, 48, and 72 h with a Zeiss Primo Vert microscope (Carl Zeiss Microscopy GmbH) connected to a Leica DFC 40 camera (Leica Microsystems AG). The total empty “wound” area (cell free) was measured using Image J software. “Wound” closure was calculated by subtracting the wound area at the indicated time periods from the initial wound area.

### Cell death and viability assays

The differential cetuximab sensitivity of colon cancer cells with stable overexpression of miR-143 or miR-145 and empty control cells, was evaluated through the assessment of cell death and cell viability. For cytotoxicity assays, HCT116 and SW48 cells were seeded in 96-well plates at 5,000 and 15,000 cells/well, respectively, and exposed to serial dilutions of cetuximab for 72 h. Cell viability was assayed using CellTiter 96 AQueous Non-Radioactive Cell Proliferation Assay (Promega, Madison, WI, USA), according to manufacturer's instructions. In brief, cell culture supernatants were replaced by 100 μL of MTS/PMS solution prepared in fresh culture media, and cells incubated at 37°C for 45 min. Changes in absorbance were measured at 490 nm, using a Model 680 microplate reader (Bio-Rad, Hercules, CA, USA). General cell death was evaluated by the Lactate Dehydrogenase Cytotoxicity Detection Kit^PLUS^ (Roche Diagnostics Gmbh, Mannheim, Germany), according to the manufacturer's protocol. Briefly, 50 μL of cell culture supernatant was transferred to a new 96-well plate, discarding the remainder cell culture supernatant. Cells attached in the well were lysed by adding 50 μL of lysis solution diluted in culture medium to obtain a cell lysate. Subsequently, 50 μL of LDH reagent mix was added to each well containing either cell culture supernatant or cell lysate, followed by a 30 min incubation at room temperature, protected from light. Absorbance was read at 490 nm, with 620 nm reference wavelengths using a Model 680 microplate reader. The percentage of LDH release was determined as the ratio between released LDH (supernatant) and the total LDH (supernatant + cell lysate), in the same well, as previously described [71].

### Real-Time monitoring of cell proliferation and viability

Dynamic proliferation and viability were monitored with xCELLigence Real-Time Cell Analyzer (RTCA) SP System (ACEA Biosciences Inc, CA, USA) and E-plates (96-well). The xCELLigence System monitors cellular events in real-time without the incorporation of labels. The system measures electrical impedance across interdigitated microelectrodes integrated on the bottom of tissue culture E-Plates. Impedance measurements allow monitorization and detection of the biological status of cells, including cell number and viability. Real-time data is provided as cell index, representing the relative change in electrical impedance that occurs in the wells [72], where increased cell number attached to the bottom of the E-plate is directly proportional to increased cell index value. For proliferation and viability assessment, 5,000 cells were seeded in each well, in 100 μl of culture medium. Twenty-four hours later, cells were incubated with cetuximab or vehicle, by adding 100 μl of cetuximab in culture media, or 100 μl of culture medium alone. Cetuximab final concentrations ranged from 0 (vehicle) to 1600 μg/ml. Cell index values were measured every 5 min, for 96 h. Cell index values from 0 to 24 h (before cetuximab incubation) were used to plot and calculate doubling times. Cell index values of cells exposed to serial dilutions of cetuximab for 72 h were used to calculate cetuximab IC_50_ values in each cell line, using RTCA Software Package (ACEA Biosciences Inc).

### Antibody-dependent cell cytotoxicity evaluation

Cetuximab- and trastuzumab-mediated ADCC was assayed using xCELLigence. Aside from viability, proliferation, and cell death, this system has been successfully used to evaluate ADCC [73–75]. For quantification of ADCC, PBMCs were used as effector cells (E). HCT116, SW480 and SW48 cells with stable overexpression of miR-143 and miR-145, and empty control cells, were used as target cells (T), HCT116 and SW480 cells were plated onto a 96-well E-plate at 5,000 cells/well and SW48 cells were plated at 15,000 cells/well, and allowed to attach and grow for 24 h. Cells were then incubated in duplicate with cetuximab or effector cells at E:T of 6:1, 10:1 and 20:1 alone, and also with cetuximab or trastuzumab together with PBMCs, and vehicle control. Cell Index was measured every 15 min up to 72 h following cetuximab or trastuzumab and/or PBMC exposure, and used to plot cell index curves. Raw cell index curves were normalized to cell index value of 1.0 at the time of treatment start. Cetuximab- and trastuzumab-mediated ADCC quantitative effects were determined by calculating area under the growth curves (AUC) during the 72 h of exposure to cetuximab or vehicle control together with effector cell exposure (AUC(72)). The percent of cell killing (Cell kill (%)) was calculated using the following formula [76]:

$$\text{Cell kill}(\%)=\frac{\begin{array}{l} \text{AUC}(72)(\text{without cetuximab})\\ -\text{AUC}(72)(\text{with cetuximab}) \end{array}}{\text{AUC}(72)(\text{without cetuximab})}\times 100$$

Cell lysis was also detected by measuring the amount of LDH released into the culture supernatant at 48 h, using the LDH assay kit. Briefly, cells were seeded at 5,000 or 15,000 cells/well for HCT116 and SW480 or SW48, respectively, and treated in the same conditions as described above. After 48 h of treatment, the plate was briefly centrifuged at 700 g, 2 min, and LDH release assay performed as described above. In brief, 50 μl of cell culture supernatant was used to determine LDH release, whereas maximum LDH release was evaluated following target cell lysis, after extensive washes with PBS to remove effector cells. Percent cytotoxicity was calculated as follows:

$$\text{Cytotoxicity}(\%)=\frac{\begin{array}{l} (\text{experimental LDH release}-\\ \text{spontaneous LDH release}) \end{array}}{\begin{array}{l} \text{maximum LDH release }-\\ \text{spontaneous LDH release}) \end{array}}\times 100$$

Where “Experimental LDH release” is the signal measured in a given treated sample, “spontaneous LDH release” corresponds to the signal measured in target cells exposed to vehicle control, and “maximum LDH release” corresponds to the signal measured in the presence of lysed tumor cells.

### Nuclear morphology and apoptosis evaluation

Apoptotic nuclei were detected using DNA-binding stain Hoechst. HCT116 cells stably expressing miR-143 or miR-145, and empty control cells, were seeded on 12-well plates over 18 mm glass coverslips, at a density of 5 × 10^4^ cells/well. Cells were exposed to 100 μg/ml cetuximab or vehicle control, PBMCs (10:1), or 100 μg/ml cetuximab with PBMCs (10:1). After 24 h of treatment, attached cells were washed with PBS to remove effector cells, fixed with 4% paraformaldehyde in PBS for 20 min, washed with PBS, and stained with 5 μg/ml Hoechst 33258 (Sigma-Aldrich) in PBS for 15 min at room temperature, protected from light. Subsequently, coverslips were washed with PBS and mounted on glass slides with Fluoromount-G (Beckman Coulter, Inc., Brea, CA). Fluorescent nuclei were evaluated by fluorescence microscopy using a Zeiss Axio Scope.A1 fluorescence microscope (Carl Zeiss Microscopy GmbH), equipped with an AxioCam HRm (Carl Zeiss Microscopy GmbH). At least five random microscopic field per sample of ~ 100 nuclei were counted for each condition. The results were expressed as the percentage of apoptotic nuclei per field.

Apoptosis was quantified using the Guava Nexin Reagent kit (Merck Millipore). The Nexin assay uses two distinct dyes, Annexin V-PE to detect phosphatidylserine (PS) on the external membrane of apoptotic cells, and the cell impermeant dye, 7-AAD, as an indicator of membrane structural integrity. For this purpose 2.5 × 10^4^ HCT116-derived cells were plated on 24-well culture plates, and treated as for Hoechst staining. After 48 h of treatment, cell culture supernatant and adherent cells were collected, centrifuged and resuspended in PBS containing 2% FBS for incubation with Guava Nexin Reagent. Subsequently, 70 μl of cell suspension were mixed with 70 μl of Guava Nexin Reagent, incubated for 20 min at room temperature, protected from light, and assayed promptly, using a Guava easyCyte 5HT flow cytometer (Merck Millipore). Sample acquisition and analysis were performed using the InCyte software module (Merck Millipore).

### Caspase-3 and -7 activity assay

Caspase-3 and -7 activation status was measured using the Caspase-Glo 3/7 Assay (Promega). In this assay, caspase-3 and -7 activity was measured evaluating aminoluciferin-induced luminescence resulting from DEVD-aminoluciferin substrate cleavage. For this purpose, HCT116 cells stably expressing miR-143 or miR-145 were seeded on 96-well plates at 5,000 cells/well, and exposed to 100 μg/ml cetuximab or vehicle control, PBMCs (20:1), or cetuximab with PBMCs (20:1). After 16 h of treatment, 75 μL of Caspase-Glo 3/7 reagent was added to each well, plates were mixed by orbital shaking for 30 s, and incubated at room temperature for 30 min. The resulting luminescence was measured using the GloMax-Multi+ Detection System (Promega).

### Total protein isolation and quantification

For isolation of total protein extracts, cells were collected and lysed using ice-cold lysis buffer (10 mM Tris-HCl, pH 7.6, 5 mM MgCl_2_, 1.5 mM KAc, 0.5% Nonidet P-40) supplemented with 1 mM DTT, Halt Protease and 1x Phosphatase Inhibitor Cocktail (Thermo Fisher Scientific) for 30 min. The lysates were then sonicated and centrifuged at 10,000 g, at 4°C, for 10 min. Total proteins in the supernatant were recovered and stored at −80°C. Protein concentrations were determined using the Bio-Rad protein assay kit according to the manufacturer's instructions.

### Immunoblotting

Steady-state levels of Bcl-2 and PARP protein were determined by immunoblot analysis. Briefly, 50 μg of total protein extracts were separated on 10 or 12% SDS-polyacrylamide electrophoresis gels and transferred onto nitrocellulose membranes. Membranes were blocked with 5% milk and incubated overnight with primary mouse antibodies reactive to Bcl-2 (#sc-7382) or primary rabbit antibody reactive to PARP (#sc-7150) (all from Santa Cruz Biotechnology, CA, USA). Membranes were then incubated with horseradish peroxidase-conjugated anti-mouse or anti-rabbit immunoglobulin secondary antibodies (1:5000 dilution; Bio-Rad). For protein detection, membranes were processed using Immobilon Western Chemiluminescent HRP Substrate (Merck Millipore) or Super Signal Femto substrate (Pierce, Rockford, IL, USA) in a ChemiDoc MP System (Bio-Rad). β-actin (#A5541; Sigma-Aldrich) was used as loading control. The relative intensities of protein bands were quantified using the Image Lab densitometric analysis program (version 4.1; Bio-Rad).

### Statistical analysis

All data were expressed as mean ± standard error of mean (SEM) from at least three independent experiments. Statistical significance was evaluated using the Student's *t* test. Values of *p* < 0.05 were considered significant.

## SUPPLEMENTARY MATERIALS FIGURES


