# Supplementary material for: miR-143 or miR-145 overexpression increases cetuximab-mediated antibody-dependent cellular cytotoxicity in human colon cancer cells
Source: Oncotarget. 2016 Jan 25;7(8):9368–87. doi: 10.18632/oncotarget.7010 (PMC4891046; doi:10.18632/oncotarget.7010)
Supplement: Supplementary file 1 [file oncotarget-07-9368-s001.pdf]

miR-143 or miR-145 overexpression increases cetuximab-mediated antibody-dependent cellular cytotoxicity in human colon cancer cells

Supplementary Materials

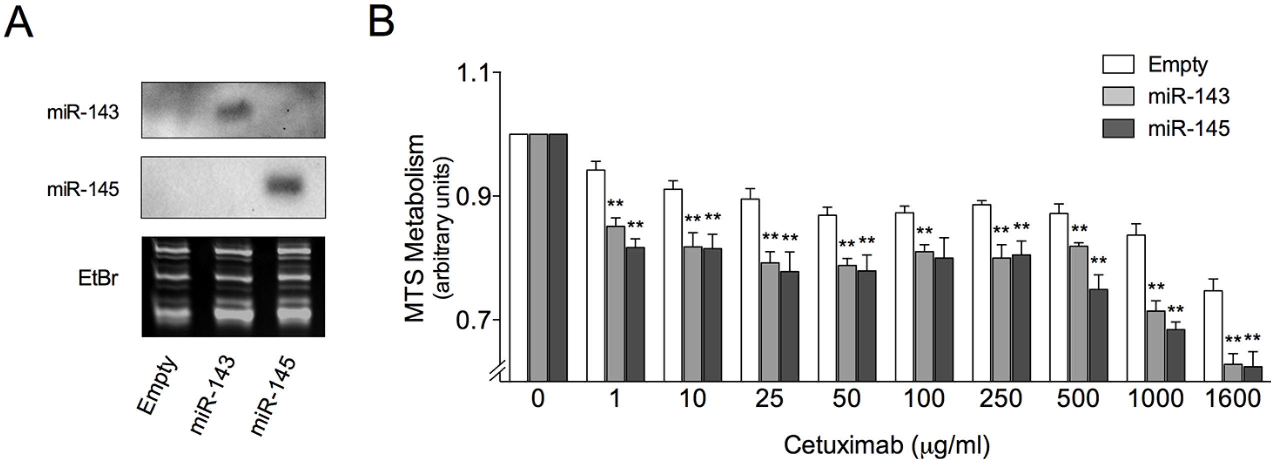

Supplementary Figure S1: miR-143 or miR-145 overexpression sensitizes SW48 wild-type KRAS colon cancer cells to cetuximab. miR-143 or miR-145 overexpressing cells were produced by transducing SW48 cell lines with viral particles containing MSCV-Neo constructs expressing miR-143, miR-145 or Empty vector, as control. (**A**) miR expression was assayed by northern blot. Gel loading controls are shown from ethidium bromide (EtBr) staining of RNA. (**B**) Cell viability was evaluated by MTS metabolism assay. The results are expressed as the mean ± SEM fold change to respective untreated cells, from at least three independent experiments. $^{**}p < 0.01$ from SW48-Empty cells.

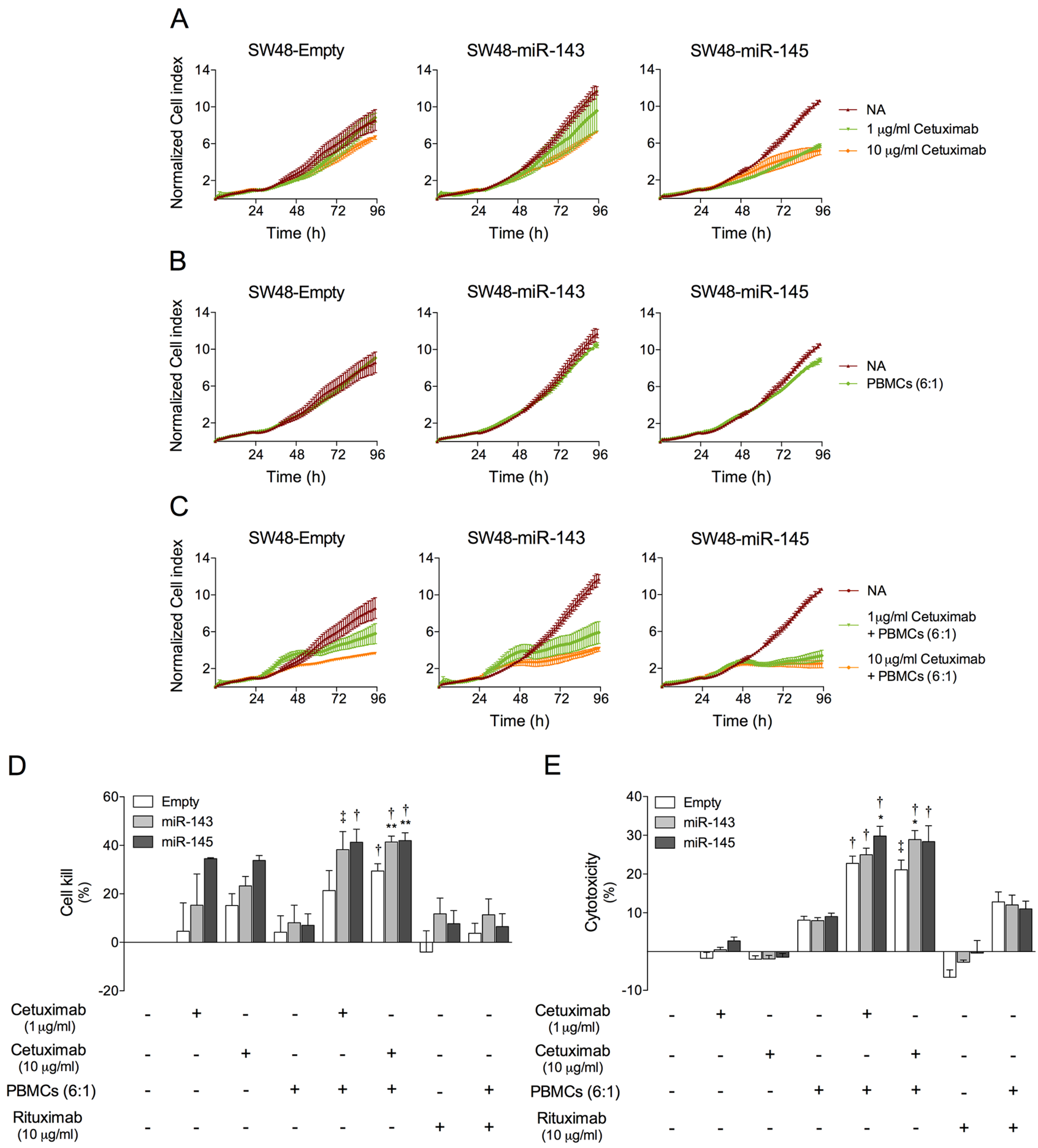

Supplementary Figure S2: miR-143 or miR-145 overexpression increases cetuximab-mediated ADCC in wild-type KRAS SW48 cells. SW48-derived cells were plated on 96-well E-Plate and used on xCELLigence System, allowed to grow for 96 h. Cells were grown in medium alone or treated with increasing concentrations of cetuximab, and or PBMCs. (**A**) Red (NA) represents cells grown in medium alone, green represents growth with 1 µg/ml cetuximab, and orange with 10 µg/ml cetuximab. (**B**) Cells were grown in medium alone (red), or treated with PBMCs at (6:1), green. (**C**) Cells were grown in medium alone (red), or treated with 1 µg/ml cetuximab and PBMCs (6:1), green, or 10 µg/ml cetuximab and PBMCs (6:1), orange. Cell index values were normalized at the time of the addition. Normalized cell index values are plotted in 1 h increments as the average of two replicates together with standard deviation. (**D**) Quantification of normalized cell index was performed at 72 h, by measuring the change in area under the curve compared to non-treated controls, and are presented as percentage of cell kill for 1 and 10 µg/ml cetuximab treatment or 10 µg/ml rituximab (control antibody), alone or with PBMCs (6:1). (**E**) Quantification of cytotoxicity was performed at 48 h, by measuring the amount of LDH released into the culture supernatant, and is presented as percentage of cytotoxicity for 1 and 10 µg/ml cetuximab treatment or 10 µg/ml rituximab (control antibody), alone or with PBMCs (6:1), compared with non-treated controls. The results are expressed as the mean ± SEM, from at least three independent experiments. $^{**}p < 0.01$ and $^{*}p < 0.05$ from respective SW48-Empty treated cells; $^{\dagger}p < 0.01$ and $^{\ddagger}p < 0.05$ from the respective cell line treated with rituximab and PBMCs.

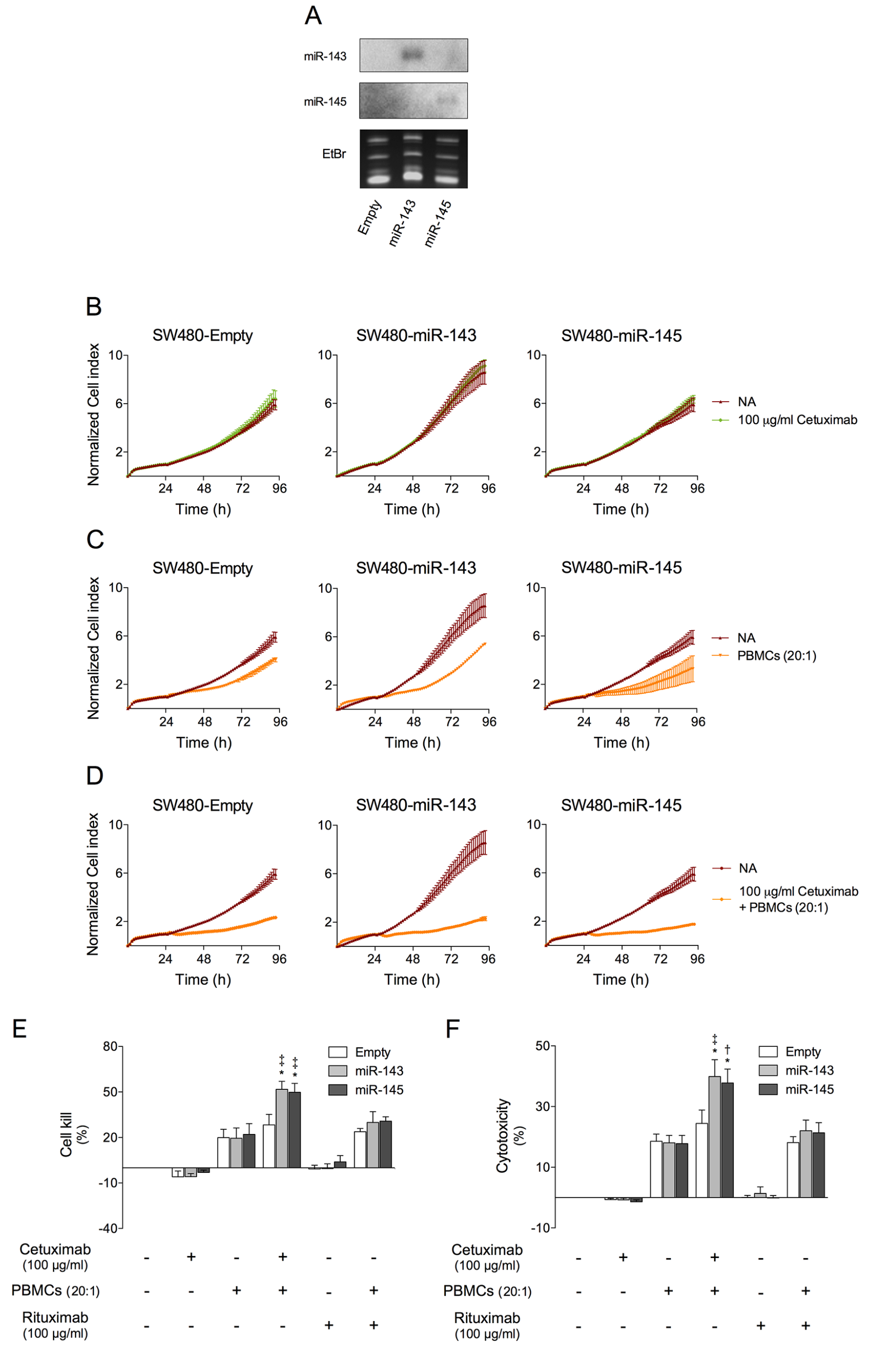

Supplementary Figure S3: miR-143 or miR-145 overexpression increases cetuximab-mediated ADCC in mutant KRAS SW480 cells. SW480 cell lines were stably traduced with viral particles containing MSCV-Neo constructs expressing miR-143, miR-145 or Empty vector, as control. (**A**) miR expression was assayed by northern blot. Gel loading controls are shown from ethidium bromide (EtBr) staining of RNA. SW480-derived cells were plated on 96-well E-Plate and used on xCELLigence System, allowed to grow for 96 h. Cells were grown in medium alone or treated with increasing concentrations of cetuximab, and or PBMCs. (**B**) Red (NA) represents cells grown in medium alone, green with 100 µg/ml cetuximab. (**C**) Cells were grown in medium alone (red), or treated with PBMCs at (20:1), orange. (**D**) Cells were grown in medium alone (red), or treated with 100 µg/ml cetuximab and PBMCs (20:1), orange. Cell index values were normalized at the time of the addition. Normalized cell index values are plotted in 1 h increments as the average of two replicates together with standard deviation. (**E**) Quantification of normalized cell index was performed at 72 h, by measuring the change in area under the curve compared to non-treated controls, and are presented as percentage of cell kill for 100 µg/ml cetuximab treatment or 100 µg/ml rituximab (control antibody), alone or with PBMCs (20:1). (**F**) Quantification of cytotoxicity was performed at 48 h, by measuring the amount of LDH released into the culture supernatant, and is presented as percentage of cytotoxicity for 100 µg/ml cetuximab treatment or 100 µg/ml rituximab (control antibody), alone or with PBMCs (20:1), compared with non-treated controls. The results are expressed as the mean ± SEM, from at least three independent experiments. $^{*}p < 0.05$ from respective SW480-Empty treated cells; $^{\dagger}p < 0.01$ and $^{\ddagger}p < 0.05$ from the respective cell line treated with rituximab and PBMCs.

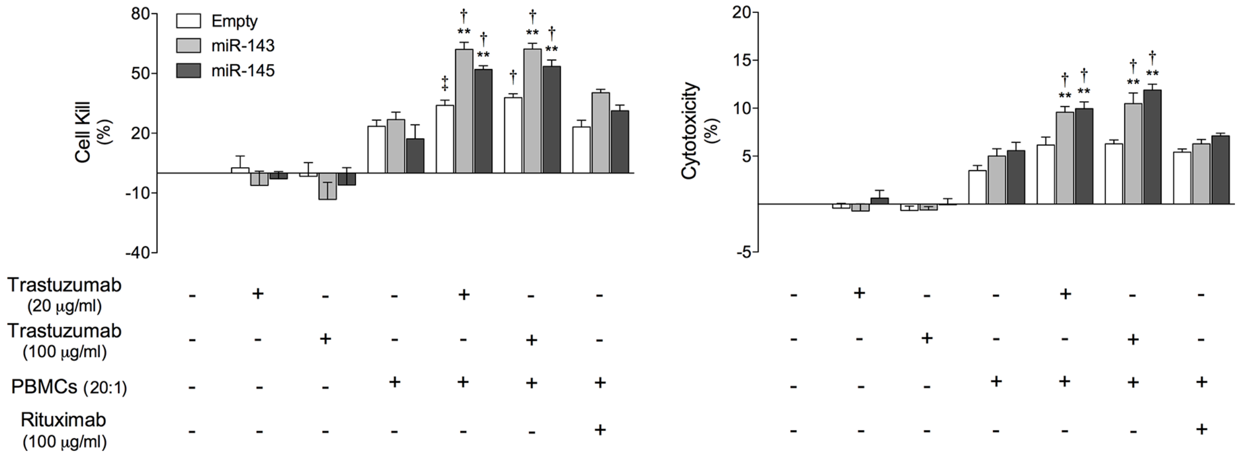

Supplementary Figure S4: miR-143 or miR-145 overexpression increases trastuzumab-mediated ADCC in HCT116 cells. HCT116-Empty, HCT116-miR-143 and HCT116-miR-145 were plated on 96-well E-Plate and used on xCELLigence System, allowed to grow for 96 h. Cells were grown in medium alone or treated with increasing concentrations of trastuzumab, and/or PBMCs. (**A**) Quantification of normalized cell index was performed at 72 h, by measuring the change in area under the curve compared to non-treated controls, and are presented as percentage of cell kill for 20 and 100 μg/ml trastuzumab treatment or 100 μg/ml rituximab (control antibody), alone or with PBMCs (20:1). (**B**) Quantification of cytotoxicity was performed at 48 h, by measuring the amount of LDH released into the culture supernatant, and is presented as percentage of cytotoxicity for 20 and 100 μg/ml trastuzumab treatment or 100 μg/ml rituximab (control antibody), alone or with PBMCs (20:1), compared with non-treated controls. The results are expressed as the mean ± SEM, from at least three independent experiments. $^{**}p < 0.01$ from respective HCT116-Empty treated cells; $^{†}p < 0.01$ and $^{‡}p < 0.05$ from the respective cell line treated with rituximab and PBMCs.
